# Effect of Fiber Fermentation and Protein Digestion Kinetics on Mineral Digestion in Pigs

**DOI:** 10.3390/ani12162053

**Published:** 2022-08-12

**Authors:** Charlotte M. E. Heyer, Neil W. Jaworski, Greg I. Page, Ruurd T. Zijlstra

**Affiliations:** 1 Department of Agricultural, Food and Nutritional Science, University of Alberta, Edmonton, AB T6G 2P5, Canada; 2 Trouw Nutrition Innovation, 3800 AG Amersfoort, The Netherlands

**Keywords:** copper, inositol phosphate, nutrient kinetics, phosphorus, pig, zinc

## Abstract

**Simple Summary:**

There is increasing interest in improving nutrient utilization in pigs and poultry and thereby reduce nutrient excretion into the environment. The present review aims to provide an overview on interactions between fermentable substrates (e.g., starch, fiber, and protein) and selected minerals on nutrient digestion and absorption to determine nutritional solutions to maximize animal performance, principally in the grower–finisher phase. Using in vitro models, the site and rate (kinetics) of nutrient digestion or fermentation of a feed ingredient or diet can be estimated. However, for minerals, no standardized methodology to assess in vitro mineral digestion exists. In vivo, the diet total tract digestibility of phosphorus might be underestimated in diets with fermentable ingredients because of increased diet-specific endogenous phosphorus losses and requires further clarification to better calculate the true total tract digestibility of phosphorus in pigs. The quantification of fiber type, composition of fiber fractions within individual raw materials, their influence on digestion kinetics, and effects on digesta pH and nutrient solubility related to fermentation should be considered. In conclusion, applications of nutrient kinetic data should be considered as part of an integrated approach to support nutrient digestion and absorption in the gastrointestinal tract of pigs, thereby helping to reduce nutrient excretion.

**Abstract:**

Nutrient kinetic data and the timing of nutrient release along the gastrointestinal tract (**GIT**), are not yet widely used in current feed formulations for pigs and poultry. The present review focuses on interactions between fermentable substrates (e.g., starch, fiber, and protein) and selected minerals on nutrient digestion and absorption to determine nutritional solutions to maximize animal performance, principally in the grower–finisher phase, with the aim of minimizing environmental pollution. For phosphorus (**P**), *myo*-inositol 1,2,3,4,5,6-hexakis (dihydrogen phosphate) (**InsP_6_**), copper (**Cu**), and zinc (**Zn**), no standardized methodologies to assess in vitro mineral digestion exist. The stepwise degradation of InsP_6_ to lower inositol phosphate (**InsP**) forms in the GIT is rare, and inositol phosphate_4_ (**InsP_4_**) might be the limiting isomer of InsP degradation in diets with exogenous phytase. Furthermore, dietary coefficients of standardized total tract digestibility (**CSTTD**) of P might be underestimated in diets with fermentable ingredients because of increased diet-specific endogenous P losses (**EPL**), and further clarification is required to better calculate the coefficients of true total tract digestibility (**CTTTD**) of P. The quantification of fiber type, composition of fiber fractions, their influence on digestion kinetics, effects on digesta pH, and nutrient solubility related to fermentation should be considered for formulating diets. In conclusion, applications of nutrient kinetic data should be considered to help enhance nutrient digestion and absorption in the GIT, thereby reducing nutrient excretion.

## 1. Introduction

In animal nutrition, an important bio-economical challenge is the parallel development of sustainable strategies to increase feed efficiency and to decrease negative effects of animal production on the environment [1]. Improvements in precision swine nutrition and environmental sustainability can be achieved in global pork production by assessing the digestion kinetics of chemical components in feedstuffs, circadian feed behavior, gastrointestinal microbiota, and functionality of feedstuffs [2]. In pig fattening, nearly 70% of production costs are related to feed [3]. Feed formulations are based on ingredient inclusion levels and their nutrient content, digestibility data, and the assumption of additivity. However, digestibility data generally do not account for interactions among nutrients or ingredients resulting in excess nutrients in diets fed to pigs [4]. Furthermore, interest exists in strategies to increase nutrient and mineral digestibility, e.g., using exogenous enzymes and thereby reducing mineral supplementation as means to reduce the environmental impact of nitrogen, phosphorus (**P**), and trace elements such as copper (**Cu**) and zinc (**Zn**) [5,6,7,8]. Nitrogen and P leaching from manure may lead to the eutrophication of fresh or seawater, with ammonia leading to acidification and eutrophication, resulting in negative effects on soil, forest, and biodiversity [5]. A further global challenge is the increasing scarcity of economically viable inorganic P sources. The main source of inorganic P (rock phosphate) is non-renewable, costly, and is geographically concentrated because six countries control 90% of the world’s phosphate rock reserves [9,10]. With only 20% of the world’s mined P being consumed by humans, the development of a sustainable resource management plan with the reduced mining of phosphate is becoming particularly important [11]. In plant materials, organic P is either present as *myo*-inositol 1,2,3,4,5,6-hexakis (dihydrogen phosphate) (**InsP_6_**), or phytate (any salt of InsP_6_). In feedstuffs, 80% of zinc is bound to InsP_6_ [7]. In piglets, supplementation of feed with exogenous phytase increases the digestibility of Zn, whereas the quantitative relationships between phytase, InsP_6_, and Zn require further investigation [7]. In a comprehensive overview, the environmental impacts of Zn and Cu used in animal nutrition were found to mainly affect groundwater, from the drainage and runoff of Zn from arable land to surface water. Copper accumulation in soil seems to be a long-term environmental concern, particularly in livestock-dense regions [12]. Agriculture and aquaculture seem to be major sources of soil and water contamination with metals such as Cu and Zn, possibly leading to accumulations triggering the co-selection of antibiotic resistance [13]. The major source of Zn emission is the land application of manure, increasing Zn levels in the top 0–20 cm layer of soil by 22–68% in the next 100 years, if current Zn inputs of different origins remain the same [14]. By reducing Zn inclusion in fattening pig diets, Zn emission could be reduced by 31%, with an additional reduction of 53% with the use exogenous or intrinsic phytases, and a further reduction in maximum Zn content in complete feed from 100 to 70 mg/kg feed [7]. Zn has been limited to 150 mg Zn/kg feed in nursery pigs since June 2022 in the EU due to risks for environmental accumulation and association with development of antimicrobial resistance [15]. In contrast, Cu amounts used for feed supplementation are usually small (0.7% of total Cu used as chemical) [16], but still represent an important source in agricultural soils where the reduction in maximum Cu content of piglet feed (from 170 down to 25 mg/kg) might support a decrease in total Cu emission by 20% [8].

In this review, the focus is on potential interactions between fermentable substrates (starch, fiber, and protein), on mineral and nutrient digestion and absorption to determine nutritional solutions to maximize performance of growing-finishing pigs, with an aim to minimize environmental pollution. Data measured in growing-finishing pigs are scarce; therefore, occasionally data from other monogastric species were included in this review. Finally, an enhanced understanding of protein and fiber digestion, and the extent of fermentation among feedstuffs, may help to increase feed formulation flexibility and producer profitability.

## 2. In Vitro Nutrient Digestion Kinetics of Feedstuffs for Pigs

Rapid and accurate feed quality assessments of digestible nutrient contents of feedstuffs are important to help avoid reduced animal performance or increased feed cost per unit of output [17]. In vivo animal trials to determine feed quality are reliable, but also expensive and time-consuming, with inherent animal welfare considerations [18].

In vitro digestion (**IVD**) and fermentation models are methods that simulate the digestion and/or fermentation processes that occur in the animals’ gastrointestinal tract (**GIT**). The IVD model assesses feed quality by mimicking natural digestion processes by directly measuring end nutrient content to estimate digestibility. Table 1, Table 2, Table 3 and Table 4 provide an overview of the classification of feedstuffs by in vitro digestion and fermentation kinetic studies using commercially available purified enzymes for all digestion and fermentation simulation steps. Although many different IVD models exist, they can be categorized in two-step IVD models simulating the stomach (step 1) and small intestine (step 2), and sometimes include a third step (step 3) mimicking disappearance of nutrients in the large intestine [18]. In vitro simulations of hindgut fermentation require inocula from living animals such as hindgut digesta or feces [18] or the use of purified enzymes (e.g., Viscozyme) [19]. The former method, however, results in digestibility values that are closer to in vivo determined values [20]. A detailed overview of methods to predict the nutritive quality of feedstuffs in vitro has been published elsewhere [18].

In addition to quantitative information on nutrient release or metabolite production, the rates (kinetics) of nutrient digestion or fermentation are important to understand the timing of dietary nutrient release along the GIT. This information could be applied to predict effects on post-absorptive appearance of nutrients such as the net portal appearance of fermentation metabolites, and related post-absorptive metabolism such as the net portal appearance of incretin and glucagon-like peptide [61]. In vitro nutrient digestion and fermentation kinetic data can then be used to optimize feed formulations using concepts such as the nutrient synchronization of energy and protein to increase nitrogen utilization in pigs [63,64,65], or modulating specific SCFA production to promote the proliferation of beneficial microbiota in the GIT [66]. As illustrated in Table 5, studies have examined digestion kinetics of starch and protein, and fiber fermentation. Furthermore, quantitative information on differences in nutrient digestion or fermentation kinetics among different feedstuffs or purified sources were classified based on fast, slow, and resistant digestible nutrients [38,39,41,61,67,68]. Nevertheless, further research into the role of the synergistic effects of nutrient digestion is required because undigested nutrients may affect the digestion of other nutrients [18]. Finally, kinetics of a single ingredient may not reflect the kinetics of a more complex ingredient blend due to ingredient interactions.

### 2.1. Starch Digestion and Fermentation Kinetics

#### 2.1.1. In Vitro Starch Digestion Kinetics

Starch digestion in the small intestine yields glucose as an end product for absorption, whereas the microbial fermentation of starch throughout the intestine produces SCFA (e.g., butyrate and propionate). Starch digestion and fermentation affect feed utilization, digestive physiology, and gut health in pigs [72]. Kinetics of starch digestion are affected by several factors, such as starch chemistry (e.g., amylose:amylopectin ratio [68]), particle size, processing method, and association with other components [73]. In pigs, IVD models have been used to determine starch digestion kinetics and glucose absorption [41]. One such IVD model mimics starch digestion by a two-step IVD process using pepsin followed by enzymatic digestion with pancreatin, amyloglucosidase, and invertase, and analysis of released glucose over time at 39 °C [72]. Based on the rate and extent of in vitro enzymatic digestion [41,67], digested fractions can be classified as rapidly digestible starch (within 20 min of incubation), slowly digestible starch (between 20 and 120 min), and resistant starch (more than 120 min, not further hydrolyzed). The in vitro glucose release was linearly related (R^2^ = 0.95) to the cumulative portal glucose appearance in pigs after correcting for predicted gastric emptying, indicating that in vitro starch digestion kinetics adequately predict net portal glucose appearance [41].

#### 2.1.2. In Vivo Application of In Vitro Kinetics

Gastric starch digestion is underestimated, consequently contributing to more rapid initial starch digestion in vivo, thereby challenging the prediction quality of in vitro assays [44]. In vivo starch digestion kinetics of nine diets differing in starch source (barley, corn, and high-amylose corn) and form (isolated, within cereal matrix, and extruded) were determined and compared with in vitro digestion values [67]. The in vivo starch digestion exceeded the in vitro predictions for rapidly digested starch. Within 5 min of small intestinal digestion in vivo, starch disappearance averaged 35% and resulted in the typical end products of α-amylase, whereas in vitro only 13% of starch was digested in the same time [44,45]. In particular, in the stomach and small intestine, the hydrolysis rate and digesta transport modulates the rates of nutrient absorption [18], whereas digesta transport is affected by several factors such as meal size [74], energy content [75], and nutrient-related mechanisms [76,77]. In addition, the substrate to enzyme ratio is also likely lower in vitro due to highly aqueous environment relative to GIT digesta. Notably, the rate of an enzyme-catalyzed reaction is proportional to the concentration of an enzyme–substrate complex according to the Michaels–Menten equation [78]. The solid and liquid digesta transport to the end of the small intestine was studied by feeding diets varying in starch source (barley, corn, and high-amylose corn) and form (isolated starch, ground cereal, extruded cereal) to pigs [79]. The mean retention time of digesta solids ranged between 129 and 225 min for the stomach and 86 and 124 min for the small intestine, with the greatest effect of dietary treatment on the solid digesta mean retention time in the stomach (extrusion reduced mean retention time by 29 to 75 min). The authors concluded that the mean retention time of stomach digesta is difficult to predict from dietary properties because of the complexity of chemical and physical digesta properties.

The amount of digested starch should be described as hydrolyzed starch (starch degradation by endogenous enzymes resulting in intermediate products, such as dextrins and glucose) for in vitro studies [68]. Accordingly, starch classification [67] should be updated into rapidly hydrolyzed starch (RHS; hydrolyzed within 20 min by pancreatin α-amylase, yielding mainly maltose and higher maltodextrins), slowly hydrolyzed starch (SHS; hydrolyzed within 20 to 120 min), and starch resistant to hydrolysis (RS_H_; not hydrolyzed within 120 min). Finally, for in vivo starch hydrolysis in pigs, starch should be classified based on the amount of starch hydrolyzed: RHS, end products of pancreatic α-amylase within 20 min after digesta enters the small intestine; RS_H_, not hydrolyzed at ileum site of small intestine; and SHS, difference between RHS and hydrolyzed starch at ileum [68].

Cereal and pulse grains differ in starch structure, amylose to amylopectin ratio [80], and where the protein matrix associated with the starch granules [81] contributes to variations in digestion kinetics in vitro [43]. An in vitro study assessed four diets differing in starch and protein sources, corn-, barley-, faba-bean-, and pea-based diets [46]. In a two-step IVD model and classifying the diets in fast, slow, and resistant starch [41], the pea-based diet had the greatest content of fast and resistant starch, possibly related to the crystalline structure and high-amylose content of pea starch granules [46]. In a growth performance trial [46], diets were fed to pigs and metabolic effects were determined by blood serum biochemical response criteria and glycemic and insulin post-prandial responses. In vivo glucose concentrations in blood 1 h after feeding were greater in pigs fed the corn-based diet than the barley-based diet, whereas the insulin concentration was greater for the barley-based diet 1 h and 2 h after feeding compared with the other treatments, possibly because soluble fiber (β-glucans in barley) affects hormonal release [82], but does not appear to affect the gastric emptying of starch [83,84]. Interactions among nutrients such as the amylose content and protein-starch bonds might be related to the metabolic response and might cause discrepancies in vitro [85]. Feeding a barley-based diet resulted in the greatest average daily gain (622 g/day) compared with other cereal and pulse grain treatments (corn, 495 g/day; faba bean 583 g/day; field pea, 581 g/day), but neither the feed intake nor final body weight were affected. The authors [46] suggested that the barley-based diet can be fed to pigs without reducing the growth performance compared with the corn-based diet. Feeding the faba bean-based diet resulted in lower blood glucose concentrations compared with the barley-based diet. Therefore, the effect of both types of starch and type of dietary fiber in ingredients affected nutrient digestion and absorption, including glycemic and insulinemic responses in pigs.

The broad picture emerging from these recent studies on starch kinetics is that the initial rate of starch digestion is greater in vivo than in vitro, resulting in faster initial starch digestion in vivo. Current studies strongly suggest that characteristics of the starch source affect the digesta retention time, particularly in the stomach. Considering that starch is quantitatively the main macronutrient in pig diets, it can be hypothesized that digestion kinetics of other nutrients, such as protein and minerals, are likely to be affected by starch characteristics as well.

### 2.2. Fiber Fermentation Kinetics

Dietary fiber comprises non-digestible carbohydrates (NSP, resistant starch, non-digestible oligosaccharides) plus lignin [86]. Fiber is poorly digested by endogenous enzymes but can be fermented by the gut microflora, affecting changes in the physicochemical properties of fiber such as the bulk, viscosity, solubility, water-holding capacity, and fermentability [72]. The rate and extent of fermentation of different dietary fiber fractions is important because the fermentation of dietary fiber mainly produces SCFA (acetate, propionate, and butyrate), lactate and gases, depending on the substrate and microbial ecology in the gut [87]. Consequently, SCFA production can be manipulated by changing substrates reaching the hindgut [88]. There is increasing interest in describing the fermentability of ingredients in the digestive tract of monogastric species to stimulate specific SCFA production through optimized diet formulations to promote beneficial microbiota with positive effects on growth performance and health [18].

The quantification of total dietary fiber (**TDF**) as the sum of the different fiber fractions (TDF = lignin, cellulose + insoluble hemicellulose + soluble hemicellulose + resistant starch + non-digestible oligosaccharides) is the first step to estimate the fermentability of a feed ingredient or diet [89]. In growing pigs, most soluble dietary fiber, such as soluble hemicellulose, appears to be fermented by the end of the cecum, whereas insoluble fiber is mostly fermented in the colon [90]. In addition, cellulose and lignin are fermented to a limited extent in the large intestine of growing pigs. In ruminant nutrition, the lignin concentration is inversely related to the rumen fermentation of ingredients and diets [91], and this may also be applicable for monogastric species such as pigs. Thus, lignin concentrations of ingredients and diets should be considered in determining fermentability.

In vitro models can be used to evaluate the rate of fermentation in the porcine digestive tract by measuring gas production and concentrations of SCFA [56,92]. This can help to estimate the location of fermentation within the pigs’ GIT, and to target beneficial effects of dietary fiber fermentation. However, in vitro models do not account for the ongoing production and absorption of SCFA that occurs in vivo. Furthermore, in vitro fermentation models need to consider a maximum length of 48 h for porcine studies [61] considering that mean retention times in other sections of the GIT can vary depending on the ingredient source [79]. Regarding starch, chemistry affects the post-ileal nutrient flow, nutrient digestibility, glucose, SCFA absorption, insulin, and incretin secretion in pigs [93,94,95]. Amylose contents affect starch hydrolysis, with high-amylose starch (“resistant” starch) having a greater resistance to enzymatic digestion [93]. Thus, resistant starch is largely fermented and enhances butyrate production in vitro [56,96,97] and in vivo [93], which may induce the growth of colonic epithelium, colonocyte differentiation, and immune responses [87]. With the in vitro gas production technique, fermentation characteristics in the hindgut of four purified starch sources differing in physico-chemical properties were studied: rapidly digestible (<45 g amylose/kg, rice starch); moderately rapid digestible (176 g amylose/kg, rice starch); moderately slow digestible (256 g amylose/kg, pea starch); slow digestible (569 g amylose/kg, corn starch) [61]. Rapidly digestible starch had the greatest fractional rate of degradation, indicating that rapidly digestible starch reaching the large intestine is fermented quickly. In contrast, slowly digestible starch is fermented at a slower rate than the other starch sources. Considering that rapidly digestible starch is digested rapidly [41], and completely [93], in the small intestine of pigs, except in young pigs [98], fermentability data and fermentation kinetics data, combined with coating technologies, could be used to specifically promote fermentation along the GIT [99].

In general, fermentable ingredients have a greater rate of degradation and produce more gas and SCFA than less fermentable ingredients [72]. The effect of treating undigested residues of corn and wheat distillers dried grains with solubles (**DDGS**) was determined with a multicarbohydrase enzyme (*Trichoderma*-based carbohydrase containing cellulase (20,000 U/kg hydrolyzed sample), xylanase (56,000 U//kg hydrolyzed sample), or in combination with protease (*Bacillus* spp. (500 U/kg hydrolyzed sample)) on in vitro fermentation characteristics using porcine fecal inoculum and the matrix structure before and after fermentation [85]. In a two-step IVD model, samples were pre-digested and their undigested residues were fermented using a mineral solution inoculated with fresh pig feces with or without enzyme supplementation. Multicarbohydrase inclusion increased fermentability (total gas and SCFA production) for corn and wheat DDGS, whereas protease in combination with multicarbohydrase inclusion reduced total gas and SCFA production and increased protein fermentation regardless of feedstuff source. The efficacy of multicarbohydrases depends on matrix porosity and ingredient source, whereas protease reduced multicarbohydrase efficacy. A later study [59] assessed the effect of supplemental xylanase (0 and 1500 U/kg diet) and mannanase (0 and 400 U/kg of diet) in an in vitro fermentation model using digested residue of corn DDGS. As a result, the addition of xylanase increased gas production after 8 h of incubation including the production of total SCFA, acetate, and propionate, indicating that supplementation with xylanase started the fermentation rapidly in the proximal part of the large intestine. The in vitro digestion and fermentation characteristics of corn wet distillers grains and corn DDGS were studied [60] without or with multi-enzyme supplementation (xylanase, glucanase, cellulase, mannanase, invertase, protease, and amylase). After a two-step IVD model, undigested residues from in vitro enzymatic digestion were evaluated using an in vitro cumulative gas-production technique. The in vitro digestibility of dry matter (**DM**) of wet distillers grain did not differ from DDGS, and multi-enzyme supplementation did not affect the in vitro digestibility of DM. However, the total gas production per unit weight of enzymatically unhydrolyzed residue was greater for wet distillers grain than for DDGS, indicting that wet distillers grain is more fermentable than DDGS. Furthermore, multi-enzyme supplementation increased total gas production for wet distillers grain and DDGS, improving the fermentability and degradation of both feedstuffs in the hindgut of pigs. Overall, there was no interaction of feedstuff and multiple enzymes on measured items, implying that drying wet distillers grain into DDGS did not affect the outcome of the multi-enzyme on digestibility of DDGS in pigs.

In monogastric species, fermentation in the GIT is important for animal health and the fermentability of ingredients can be used in formulating diets to stimulate beneficial microbial activity in the GIT [18]. In vitro gas and SCFA production have been used to evaluate wheat bran, soybean hulls, corn bran, oat bran, and sugar beet pulp [62]. The fermentation of wheat bran and oat bran resulted in a higher and faster gas and SCFA production compared with corn bran, sugar beet pulp, and soybean hulls, and the effects were positively correlated with TDF fractions of ingredients. However, the in vitro fermentation responses differed in microbial composition and SCFA production among ingredients. Thus, in vitro fermentation studies can not only provide information on gas and SCFA production, but also microbial composition contributing to the enhanced utilization of fibrous ingredients fed to pigs. Assessing the fermentation kinetics of feedstuffs or diets is a promising approach; however, further studies are required to incorporate kinetic information into formulation practices.

### 2.3. Protein Digestion Kinetics

To predict crude protein (**CP**) and AA digestibility, a two-step IVD model is often used. The in vitro digestibility of CP in feedstuffs is considered reliable for calculating coefficients of apparent ileal digestibility (**CAID**) of individual AA [31]. However, the validation of protein IVD models seems less satisfactory regarding predicted accuracy, especially for the standardized ileal digestibility of amino acids. The IVD models thus require improvement to better predict protein and AA digestibility accurately [18]. In humans [100,101], protein sources with comparable ileal protein digestibility differed in protein digestion kinetics, modulating postprandial appearance of AA and peptides in blood, and post-absorptive metabolism. In adult humans [100], postprandial AA appearance in blood was earlier for fast-digestible whey protein than slow-digestible casein. Similarly, in young men, postprandial retention was better for slowly digested casein than rapidly digested whey proteins [101].

In general, protein digestion kinetics depend on the chemical composition, protein structure, and physicochemical properties of feedstuffs [38]. The ANF may modulate the digestion and utilization of dietary protein and AA. Effects of thermomechanical and enzyme-facilitated processed SBM compared with non-processed SBM on in vitro kinetics of protein digestion and protein and AA digestibility in weaned pigs have previously been studied [39]. Processing reduced the ANF content (lectin, trypsin inhibitor activity, β-conglycinin, and glycinin) compared with non-processed SBM, and increased digested CP, tended to increase fast-digestible CP, and reduced slow and resistant CP compared with non-processed SBM. In addition, CAID and the standardized ileal digestibility of CP and of most AA were greater than in non-processed SBM indicating that processing shortened the time of digestion, increased the extent of digestion, and possibly reduced the risk of protein fermentation in the large intestine.

In broiler chickens, in vitro and in vivo protein digestibility assays can predict the rate and extent of digestion of ingredients [36,37,102]. Evidence exists that the site and rate of the digestion of protein and the absorption of AA affect broiler performance [103,104,105]. Broiler chickens were fed a protein source with either a rapid or slow protein digestion rate and two dietary fiber sources (oat hulls or sugar beet pulp) to assess the effects on growth performance [105]. Broilers fed diets containing rapidly digestible protein had greater average daily gain (**ADG**) and feed efficiency (gain:feed) after the starter phase. In this study, the ADG (day 28–36; day 0–36) and feed efficiency (day 28–36) of broilers fed slowly digested protein diets with oat hulls did not differ from rapidly digested protein diets supplemented with sugar beet pulp or oat hulls. However, the addition of insoluble dietary fiber, such as oat hulls, to slowly digestible protein could improve performance to the level of broilers fed costly rapidly digested protein, probably by increasing the rate of digesta passage through the distal GIT, resulting in greater feed intake. Effects of feedstuffs (corn, wheat, sorghum, soybean meal, canola meal, full-fat soybean, palm kernel meal, meat and bone meal, wheat DDGS, and wheat bran) on nitrogen and starch digestion kinetics were assessed in broiler chickens [106]. Overall, starch digestion kinetics were faster than nitrogen digestion kinetics. For nitrogen, the disappearance rate was affected by feedstuff, and interactions among feedstuffs, such as for full-fat soybean meal and soybean meal, decreased the nitrogen digestion rate by 25% compared with diets with only soybean meal or full-fat soybean meal. The authors concluded that knowledge about nutrient digestion kinetics and the additive and non-additive effects of feedstuffs in complex diets are important. The transition of dietary protein and amino acids into carcass protein in broiler chickens, and strategies to enhance this transition including nutrient digestion kinetics, were reviewed comprehensively elsewhere [107].

## 3. Utilization of Selected Minerals in Pigs

### 3.1. Digestibility of Phosphorus in Pigs

The porcine small intestine, in particular the jejunum, is the major site of P absorption [108]. In general, P homeostasis is regulated by controlling the absorption rate of inorganic phosphate in the upper small intestine and by renal phosphate excretion orchestrated mainly by parathyroid hormone and calcitriol (1,25-dihydroxycholecalciferol; 1,25-(OH)_2_D_3_) [109]. In addition to the absorption capacity of the pigs’ intestine, differences in dietary P digestibility must be considered because grains and their co-products are major ingredients in pig diets. Indeed, InsP_6_ is the most important source of organic P for pigs, whose dietary content ranges between 2 and 3 g/kg DM in diets for pigs and poultry depending on the ingredients, agronomy, and processing conditions [110]. In addition, InsP_6_ is considered an anti-nutritional factor (**ANF**) forming complexes with minerals and decreasing the absorption of cations and protein in pigs and poultry [111]. The hydrolysis of InsP_6_ is incomplete in non-ruminants because of insufficient endogenous phytase activity in the proximal GIT [112], and depends on several factors such as the intrinsic phytase activity of dietary ingredients, endogenous mucosal, and gut phytase activity [110]. The hydrolysis of InsP_6_ can be increased by supplementing microbial phytase to diets to increase the coefficient of apparent total tract digestibility (**CATTD**) of P by 26% to 65% [113]. However, studies with pigs focusing on the stepwise degradation of InsP_6_ to lower inositol phosphate (**InsP**) forms in the GIT have been rare [110], and just a few studies [113,114,115] differentiated positional InsP forms. A recent study [113] determined that pigs fed a corn–soybean-meal-based diet with up to 3000 FTU exogenous *E.coli*-derived 6-phytase/kg feed (Experiment 1) or a corn–soybean meal or a corn–soybean meal-rapeseed cake diet supplemented with 1500 FTU/kg feed (Experiment 2), had greater concentrations of Ins(1,2,5,6)P_4_ and lower concentrations of inositol phosphate_5_ (**InsP_5_**) isomers (Ins(1,2,3,4,5)P_5_; Ins(1,2,4,5,6)P_5_) in ileal digesta (Experiment 1) for diets supplemented with microbial phytase (1500 and 3000 FTU/kg feed). The Ins(1,2,5,6)P_4_ was considered the limiting isomer of InsP degradation for the used microbial phytase, in agreement with previous studies [116,117]. Thus, to increase the digestibility of dietary plant P, strategies to degrade InsP_6_ to lower forms of InsP are warranted because lower InsP forms can almost be completely digested by pigs [113].

Net P absorption from the hindgut of pigs is extremely limited; thus, CAID and CATTD of P do not differ widely [118]. In pigs, most InsP_6_ reaching the porcine hindgut is almost completely hydrolyzed (CATTD InsP_6_, 0.99) by endogenous phytases, probably of bacterial origin, when feeding diets based on corn and soybean meal or corn, soybean meal, and rapeseed cake. Dietary CATTD of P was the greatest at 0.64 for the corn–soybean meal diet supplemented with 1500 FYT phytase/kg diet, suggesting that the released P in the hindgut was not utilized by the pig, but excreted [113]. Endogenous secretion of P into the intestinal tract of pigs that is not absorbed is considerable [119]. The CATTD of P is not additive in pigs [120], whereas the coefficients of standardized total tract digestibility (**CSTTD**) or true total tract digestibility (**CTTTD**) of P in feed ingredients, when corrected for basal or basal and diet-specific endogenous P losses (**EPLs**), are favorable (i.e., additive) for diet formulation. Adequate correction of EPL is important because basal EPLs measured using a P-free diet varied greatly, ranging from 129 to 219 mg P/kg DMI [121]. The total EPLs of P estimated using regression (8 to 455 mg P/kg DMI) varied greatly [119]. The EPLs are diet-dependent and increase with increasing contents of dietary NSP, and may depend on dietary fiber properties [122,123]. In growing pigs, the effect of increasing the dietary inclusion of acacia gum, a low-viscous, fermentable fiber, on nutrient digestibility was assessed in growing pigs fed a low-P control diet (to measure basal EPL), and three additional diets including 25, 50, or 75 g/kg as-fed acacia gum at the expense of corn starch [124]. Increasing the inclusion of acacia gum tended to linearly increase the total tract EPL (basal EPL 377 mg/kg DM intake), likely due to the greater excretion of P of bacterial origin, increased epithelial cell proliferation rate, and sloughing of epithelial cells [125,126]. Moreover, increasing the inclusion of acacia gum tended to linearly decrease diet CAID and CATTD of P, and CSTTD of P calculated based on measured EPL or applying NRC’s [4] recommended value (190 mg P/kg DM intake), likely because of the increased diet-specific EPL. Specific EPLs associated with feeding ingredients high in low-viscous fermentable fiber require further clarification to better calculate CTTTD of P, thus avoid underestimating dietary P digestibility.

### 3.2. Mineral Digestion Kinetics

As summarized in Table 3, few studies have examined mineral digestibility In vitro, and studies on mineral kinetics are limited to Ca, InsP_6_, P, and Zn. For other minerals (e.g., Mg, Cu, and Fe), kinetic studies have not been conducted; consequently, IVD models could be developed to determine the rate and extent of mineral digestion to further optimize diet formulations and decrease Cu and Zn emissions. For P and InsP_6_, no standardized methodology to assess in vitro digestion exists, in particular including phytase supplementation [127]. In vitro assays for non-ruminants focusing on phytase and phytate have been comprehensively reviewed [127].

The digestibility of P differs between plant- and animal-based feedstuffs. In vitro P digestibility was measured in 10 plant-based (alfalfa meal, barley, canola meal, corn, grain sorghum, oats, rice bran, SBM, wheat, and wheat bran) and 4 animal-based feedstuffs (menhaden fish meal, meat and bone meal, spray-dried blood meal, and dried whey) [49]. The in vitro data of the plant-based feedstuffs correlated with in vivo P digestibility (R^2^ = 0.72–0.88), whereas the animal-based feedstuffs were poorly correlated (R^2^ = −0.26–0.70). After modification, the IVD model was validated against in vivo digestibility in growing pigs fed diets based on wheat, barley, corn, potato protein concentrate, soybean expeller, or rapeseed expeller [54]. The IVD model accurately predicted the CATTD of P in plant-based diets (R^2^ = 0.91) and is an inexpensive model to rapidly estimate the CATTD of P in plant feedstuffs. However, further improvements should focus on applied digestive enzymes and the time of digestion modulation using in vitro P digestion models [128], and in improving the prediction of P digestibility of animal byproducts.

#### 3.2.1. Fiber and Mineral Digestibility

Observed effects of dietary fiber on the digestion, absorption, and utilization of minerals in pigs are not consistent [126]. In rats, the effect of Ca on inulin fermentation in the large intestine was assessed by feeding a basal fiber-free, wheat starch-casein diet, or a basal diet supplemented with 150 g/kg chicory inulin, and differing Ca content [129]. Caecal pH was lower, and mineral solubility and Ca absorption were greater in rats fed the diets supplemented with inulin compared with the fiber-free diet. The effects of feeding cellulose, corn starch, and pectin in a low-P basal diet on mineral digestibility were assessed in pigs [130]. The CAID of Ca and CATTD of P and Ca in diets were lower for the pectin diet than basal diets, leading to the conclusion that carbohydrate source affected inevitable P losses. In piglets fed barley–wheat–SBM diets supplemented with potato fiber (50 g/kg) or lignocellulose (20 g/kg), CATTD of P and Zn were greater for the potato fiber than with the lignocellulose diet, whereas CATTD of InsP_6_ was lower for the potato fiber than the lignocellulose diet [131]. In contrast, CAID and CATTD of P in diets supplemented with different dietary fiber sources (pectin, cellulose, straw meal, inulin) did not differ [132,133]. In this regard, differences in the content and properties of dietary fiber and InsP_6_ contents of ingredients must be considered to improve predictions on the effects on digesta pH and nutrient solubility affecting mineral digestibility.

**Figure 1:**
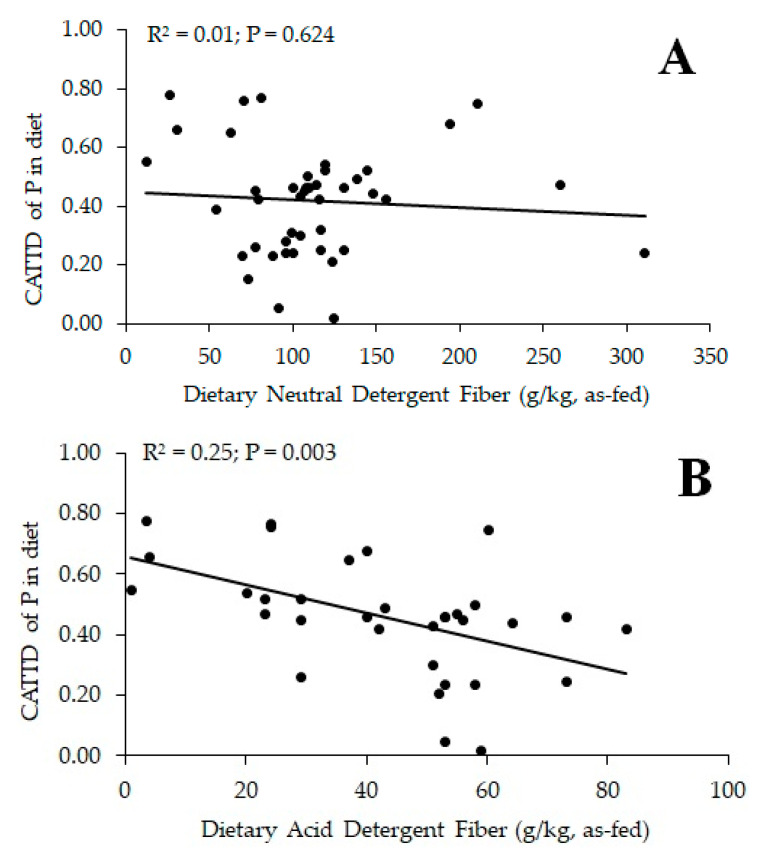
Relationships between diet neutral (**NDF**; **A**) or acid detergent fiber (**ADF**; **B**) content and coefficient of apparent total tract digestibility (**CATTD**) of phosphorus (**P**) in diets fed to pigs using data in Table 8 and Table 9 [115,130,131,132,135,137,138,147]. Data were analyzed using PROC REG of SAS (version 9.4; SAS Institute) to determine linear relationships between diet NDF or ADF content and CATTD of P in diets. A value of *p* < 0.05 was considered significant.

Researchers have observed contradictory effects of dietary fiber on P digestibility in monogastric species; thus, the effect of type and inclusion level of dietary fiber should be considered (Table 6, Table 7, Table 8 and Table 9). In growing pigs fed corn-starch-based diets differing in ingredient compositions (SBM, corn DDGS, and CM) at three inclusion levels [134], CAID and CATTD of P were linearly increased by increasing SBM at the expense of corn starch, but there was no effect of increasing corn DDGS or CM inclusion in diets. The effects of feeding barley grain cultivars differing in amylose, β-glucan, and fiber content on mineral digestibility were compared with wheat [135]. Moderate- and low-fermentable barley, and low-fermentable wheat had greater diet CAID of P than highly fermentable barley. In addition, moderate-fermentable barley had greater diet CATTD and CSTTD of P than highly fermentable, high-β-glucan barley, concluding that cereal grains high in fermentable (slowly digestible) fiber (e.g., β-glucans) in specific hull-less barley cultivars resulted in lower dietary CAID, CATTD, and CSTTD of P. In contrast, CATTD of P did not differ between corn starch-casein diets with or without 89.5 g/kg as-fed oat β-glucan concentrate at the expense of corn starch [136]. In growing pigs, CATTD and CSTTD of Ca and CATTD of P fed in a corn-based diet were greater than in a corn-starch-based diet and added phytase and fiber (80 g/kg cellulose at the expense of corn starch) increased CATTD and CSTTD of Ca and CATTD of P in diets [137]. Tail-end dehulling of canola meal that constituted largely removal of insoluble fiber, increased CATTD and CSTTD of P in growing pigs [138]. In a two-step IVD model [139], Zn digestibility was greater in low-InsP_6_ whole pearl millet flour, and decorticated low-InsP_6_/fiber/tannin pearl millet fractions than in high-fiber/tannin bran fractions and high-InsP_6_ decorticated fractions. However, Zn digestibility was greater for low and high InsP_6_ bran fractions than in high-InsP_6_ decorticated fractions, possibly because of the lower InsP_6_:Zn molar ratio. In piglets, the effect of Zn source and dietary fiber (lignocellulose, potato fiber) on nutrient digestibility were assessed. The CATTD of P and Zn were greater, whereas CATTD of InsP_6_ in diets was lower for potato fiber than lignocellulose diets [131]. The CATTD of P in diets was linearly related to the ADF content of the diet, reducing CATTD of P in diets with increasing ADF content, but not to the dietary NDF content (Figure 1). Lignin concentrations might reduce fermentation in monogastric species, similar to that observed in ruminants [91]. The estimation of fermentability of feed ingredients or diets based on TDF [89] should be considered in determining the CATTD of P. Nevertheless, further research into the role of dietary fiber and minerals is still required, with special focus to be directed to individual InsP on mineral digestibility. InsP_6_ and the chemical structure of individual NSP affect digestion and fermentation of nutrients; therefore, future research should concentrate on ingredient and feed processing strategies that increase nutrient digestibility and animal performance, such as pre-digestion techniques using exogenous enzymes [111,140].

#### 3.2.2. Nutrient Kinetics and Exogenous Enzymes

In vitro, one part of InsP_6_ in cereals and oilseeds is readily degraded, whereas the residual part requires a longer digestion time [148]. The less degradable part of InsP_6_ may consist of complexes formed with minerals and/or proteins, or InsP_6_ may be encapsulated within the cell wall matrix, reducing its accessibility to exogenous enzymes such as phytase [50,53,149]. Using an IVD model simulating the fish stomach, effects of *E. coli* phytase (2500 FYT/kg DM) supplementation on P and protein digestibility in eight plant ingredients (SBM, field pea, broad bean meal, chickpea protein isolate, lupin meal, canola meal, wheat middlings, and wheat flour) was assessed [150]. Degradation of InsP_6_ by exogenous phytase increased protein solubility in all ingredients, except wheat flour (pH-dependent: broad bean meal, field peas, acidic and neutral pH; SBM, chickpea protein isolate, acidic pH; lupin meal, pH 4.0, 5.0; wheat middlings, pH 2.0, 3.0, 4.0, 5.0; canola meal, pH 2.0, 3.0). Exogenous phytase might increase protein solubility in leguminous seeds, but to a lesser extent in cereals and canola meal. Such conclusions are in agreement with a broiler study feeding seven plant ingredients (corn, SBM, wheat, wheat middlings, barley, defatted rice bran, and canola) as the sole source of P with and without added phytase (600 FYT phytase/kg diet [151]). Total tract degradation of InsP_6_ was greatest for the SBM and barley diet and lowest for the wheat and defatted rice diet. In broilers, addition of exogenous *Buttiauxella* phytase (500 FYT/kg feed) to a corn-based diet increased proximal jejunal, distal ileal starch, proximal, distal jejunal and ileal protein apparent digestibility, and reduced starch:protein disappearance rate ratios [152]. Exogenous phytase inclusion increased the apparent AA digestibility in four intestine segments, particularly the proximal jejunum. In addition, the apparent digestibility of Na and P was greater for phytase-supplemented diets in the proximal and distal small intestine, whereas Na CAID was correlated with starch and protein CAID. Increasing protein disappearance rates in the proximal ileum would be advantageous, whereas increasing starch disappearance rates would be disadvantageous in terms of weight gain over 40 days, emphasizing that starch and protein digestion kinetics and the post-enteral availability of glucose and AA at sites of protein synthesis are important for broiler growth. The authors concluded that supplementation with exogenous phytase and the effect on Na apparent digestibility in the small intestine might be relevant for glucose and AA absorption.

Phytate sources (phytate content, location, and ingredient matrix) might determine the extent of InsP_6_ degradation [127]. Moreover, the degradation of InsP is reduced by increasing the dietary Ca content in monogastric species [153]. Increasing pH [154] and small calcium carbonate particle size (28 µm) with greater solubility (>70%) increased Ca–phytate complex formation or inhibited phytase efficacy [127,155].

In rats, Zn forms insoluble complexes with phytate in the GIT, reducing Zn digestibility [156,157]. A two-step IVD model was applied to estimate the Ca, Mg, Fe, Cu, and Zn digestibility of eight different breads (white, brown, whole meal wheat, rye, brown bread with sunflower seed, white bread with hazelnut, sourdough fermented brown, and sourdough fermented brown bread with sunflower seed) varying in InsP_6_ content [158]. During pancreatic digestion, the in vitro digestibility, measured as dialysability, was decreased for Ca, Mg, Fe, and Cu with increasing pH (from 6.6 to 7.1), whereas the digestibility of Zn was not affected, suggesting a strong effect of pH on mineral digestibility. In addition, the InsP_6_ content of the breads might have reduced the digestibility of Ca, Fe, and Zn, possibly by forming insoluble Ca–Zn–phytate complexes with increasing pH [159]. Effects of dietary Zn source included in corn–SBM-based diets differing in Zn, phytate, and exogenous phytase (500 FYT *Aspergillus niger* phytase/kg feed) content were assessed in piglets [160]. Phytase supplementation increased soluble Zn in the stomach and tended to increase soluble Zn content in the intestine, possibly by lowering gastric pH and resulting in increased mineral solubility [145,161].

In grower pigs, the effects of wheat millrun inclusion (200 or 400 g/kg), xylanase (0 or 4375 U/kg feed), and phytase (0 or 500 U/kg feed) level on nutrient digestibility and growth performance were assessed [142]. The CAID and CATTD of P and Ca were reduced linearly with the increasing inclusion of wheat millrun. The supplementation of xylanase increased CAID of P in diets. There was a synergistic effect of xylanase and phytase increasing CATTD of P, resulting in a similar CATTD of P for the 200 g/kg wheat millrun than in the wheat control diet. The authors concluded that NSP and phytate limit nutrient digestibility in wheat co-products, and exogenous phytase and xylanase supplementation increased P digestibility. The effect of the dietary fiber content of rapeseed meal with and without added microbial phytase (*Aspergillus niger* 500 FYT/kg feed) on mineral digestibility in growing pigs was studied [147]. Feeding exogenous phytase increased CATTD of P, and gastric and cecal inorganic P solubility was greater in pigs fed diets with exogenous phytase. The dietary inclusion of 45 or 90 g/kg rapeseed hulls quadratically increased inorganic P solubility in the caecum. The decrease in digesta pH from the distal ileum into the cecum increased inorganic P solubility and might increase P absorption. In the caecum, dietary fiber might affect nutrient solubility by increasing the effect of microbial phytase on P digestibility.

The digestibility of P for other co-products, such as DDGS, is enhanced by the fermentation process lowering the phytate content, resulting in a greater CATTD of P of wheat–pea-based diets with corn-, wheat-, or corn–wheat DDGS than in wheat–pea diet [144] without DDGS. Similarly, the CATTD of P was 60% lower for the wheat diet than wheat–DDGS diet and was not affected by supplementation with 4000 U/kg feed xylanase [143]. Feed processing, such as the fermentation of wheat bran, resulted in greater dietary CATTD of P than feeding untreated or extruded wheat bran, probably because of a lower phytate content in fermented wheat bran diets [146]. Further research into the effects of feed processing technologies on nutrient and mineral digestion kinetics is warranted.

## 4. Conclusions

Current feed formulations for pigs and poultry are largely based on ingredient inclusion levels, nutrient level constraints, and nutrient digestibility data to increase feed efficiency and reduce nutrient excretion. However, the rate and extent of nutrient digestion or fermentation, and information about the timing of dietary nutrient release along the GIT, are missing. Nutrient kinetic data could be applied to help predict effects on post-absorptive nutrient appearance, and to further improve nutrient utilization and diet formulation practices. Novel approaches such as the combination of in vitro and in silico methods for feedstuff evaluation to predict in silico digestive processes such as digesta transit, nutrient hydrolysis, and absorption kinetics of feedstuffs can be used to evaluate the extent of nutrient digestion of feedstuffs in the GIT [162].

Although results on the interaction between minerals and fermentable substrates are inconsistent, several studies have shown that CSTTD of P of diets might be underestimated in diets with fermentable ingredients because of increased diet-specific EPL; thus, the CTTTD of P should be used. The quantification of TDF (and/or other fiber fractions) to estimate fermentability should be considered when formulating diets to influence digesta pH and nutrient solubility, and to predict mineral and protein digestibility. Due to differences in P digestibility, and the formation of individual InsP because of variations in InsP_6_ content, the intrinsic and exogenous phytase effects of feed ingredients should be considered. Nevertheless, further research regarding standardized methodologies to assess in vitro digestion is still required, in particular to improve the use and application of digestive enzymes and time of digestion, but also on the effects of undigested nutrients on digestive processes.

## Figures and Tables

**Table 1:** Classification of undigested feedstuffs or diets by in vitro protein digestion kinetic studies using commercially available purified enzymes for all simulation steps.

In Vitro Model		Feedstuff	Items	Reference
Enzymes	Sample Points (min)			
Pepsin (24 h); trypsin (24 h)	1440 (24 h)	Soybean protein isolate	CP	[21]
Pepsin (48 h) or pepsin (24 h) and trypsin (48 h)	2880 (48 h)	SBM	CP, AA	[22]
Pepsin (90 min); pancreatin, amylase (60 min)	60	7 feedstuffs, 16 diets	CP	[23]
Pepsin (90 min); pancreatin, α-amylase (60 min)	60	5 common feedstuffs	CP	[24]
Pepsin (6 h), pancreatin (18 h)	1080 (18 h)	15 common feedstuffs	CP, AA	[25]
Pepsin, pancreatin, Viscozyme	–	Barley, hulled and hull-less	Gross energy, CP	[26]
Pepsin (6 h); pancreatin (18 h); cellulase (24 h)	1440 (24 h)	Barley, hulled and hull-less		[27]
Pepsin (24 h); pancreatin (96 h)	5760 (96 h)	Animal protein	Protein, peptides	[28]
Pepsin without/with phytase	0, 30, 60, 120, 180, 240	SBM extract	CP	[29]
Pepsin (6 h), pancreatin (18 h)	1080 (18 h)	Barley, 7 samples year A, 11 samples year B	OM, CP, AA	[30]
Pepsin (6 h), pancreatin (18 h)	1080 (18 h)	Ingredients, diets	CP, AA	[31]
Pepsin	10, 30, 60, 120, 240	Grower diet	CP	[32]
Pepsin (120 min); pancreatin, protease, amylase, lipase (240 min); Viscozyme (8 h)	Pepsin: 5, 10, 20, 30, 60, 120; pancreatin, protease, amylase, lipase: 10, 20, 30, 60, 90, 120, 180, 240; Viscozyme: 10, 20, 30, 60, 120, 180, 240, 480	Wheat, barley, wheat bran, SBM	OM, nitrogen, starch	[33]
Pepsin (240 min); pancreatin (240 min)	240	Fibrous diets	OM, CP, starch	[34]
Pepsin (75 min); pancreatin (210 min)	210	Nursery diets	CP, AA	[35]
Pepsin (30 min); pancreatin (180 min)	0, 15, 30, 45, 60, 90, 120, 150, 180	SBM, corn gluten meal, corn DDGS, porcine meal, fish meal, casein	CP	[36]
Pepsin (30 min); pancreatin (180 min)	0, 15, 30, 45, 60, 90, 120, 150, 180	SBM, corn gluten meal, corn DDGS, fish meal, canola meal, meat and bone meal, feather meal, blood meal	CP	[37]
Pepsin (90 min); pancreatin; bile solution	0, 30, 60, 90, 120, 150, 180, 210	SBM, wheat gluten, rapeseed meal, whey powder, dried porcine plasma protein, yellow meal worm larvae, black soldier fly larvae	Low-molecular-weight peptides (<500 Da), nitrogen	[38]
Pepsin; pancreatin; bile extract	0, 30, 60, 120, 240, 360	SBM, SBM; thermomechanical, enzyme-facilitated	CP	[39]

Abbreviations: AA: amino acid; CP: crude protein; OM: organic matter; SBM: soybean meal. Viscozyme is a mixed multi-enzyme complex containing a wide range of microbial carbohydrases including arabinose, cellulase, β-glucanase, hemicellulose, xylanase, and pectinase.

**Table 2:** Classification of undigested feedstuffs or diets by in vitro starch digestion kinetic studies using commercially available purified enzymes for all simulation steps.

In Vitro Model		Feedstuff	Items	Reference
Enzymes	Sample Points (min)			
Pepsin (30 min); pancreatin, amylase, amyloglucosidase, invertase	20, 120, 150, 180, 210, 240, 300, 360, 420, 480	Barley, extruded barley, field pea, extruded field pea, potato starch and wheat bran mixture, extruded potato starch and wheat bran mixture	Starch, glucose	[40]
Pepsin (120 min); pancreatin, protease, amylase, lipase (240 min); Viscozyme (8 h)	Pepsin: 5, 10, 20, 30, 60, 120; pancreatin, protease, amylase, lipase: 10, 20, 30, 60, 90, 120, 180, 240; Viscozyme: 10, 20, 30, 60, 120, 180, 240, 480	Wheat, barley, wheat bran, SBM	OM, N, starch	[33]
Pepsin (240 min); pancreatin (240 min)	240	Fibrous diets	OM, CP, starch	[34]
Pepsin (30 min incubation); pancreatin; amyloglucosidase; invertase	0, 15, 30, 60, 120, 240, 360, 480	Rice starch (<50 g/kg DM amylose), rice starch (196 g/kg DM amylose), pea starch (284 g/kg DM amylose), corn starch (632 g/kg DM amylose)	Glucose	[41]
Pepsin (30 min incubation); pancreatin; amyloglucosidase; invertase	0, 15, 30, 60, 90, 0, 120, 180	Corn-, pea-, rice-starch-, and white-bread-based diet	Starch	[42]
Pepsin (30 min), pancreatin, amyloglucosidase, invertase	0, 15, 30, 60, 120, 240, 360	15 different starches; one additionally sieved in 5 fractions	Starch, glucose	[43]
Pepsin (30 min), pancreatin, amyloglucosidase, invertase	0, 15, 30, 60, 120, 240, 360	9 diets differing in starch source (barley, corn, high-amylose corn); form (isolated starch, ground cereal, extruded cereal)	Starch, glucose	[44]
Pepsin (15 min), enzymes extracted from stomach digesta, enzymes porcine saliva	0, 22.5, 45, 67.5, 90, 112.5, 135, 157.5, 180, 202.5, 225	3 diets with only starch from barley origin (isolated barley starch, ground barley, extruded barley)	Glucose, maltodextrin	[45]
Pepsin (30 min); pancreatin, amyloglucosidase, invertase	0, 20, 120	Corn- barley-, faba-bean-, pea-based diet	Glucose	[46]

Abbreviations: CP: crude protein; OM: organic matter; SBM: soybean meal. Viscozyme is a mixed multi-enzyme complex containing a wide range of microbial carbohydrases including arabinose, cellulase, β-glucanase, hemicellulose, xylanase, and pectinase.

**Table 3:** Classification of undigested feedstuffs or diets by in vitro mineral digestion kinetic studies using commercially available purified enzymes for all simulation steps.

In Vitro Model		Feedstuff	Items	Reference
Enzymes	Sample Points (min)			
Phytase	0–1020	Wheat bran, whole flour of rye, whole flour of oats	InsP_6_-InsP_3_	[47]
Pepsin; exogenous phytase (0, 250, 500 FYT/kg) (75 min); pancreatin	60, 120, 180, 240, 300	Corn-SBM diet	P	[48]
Pepsin, acid phosphatase (75 min); pancreatin	0, 240	Alfalfa meal, barley, canola meal, corn, grain sorghum, oat, rice bran, SBM, wheat, wheat bran, fish meal, meat and bone meal, spray-dried blood meal, dry whey	P	[49]
Phytase	0–1380 (23 h)	Canola meal	InsP_6_	[50]
[15 min incubation, pH 6.0]; phytase; phosphatase or mixture of equal dose	0, 60, 120, 180, 240, 300, 360	Barley, corn, SBM	Orthophosphate	[51]
Pepsin without/with trypsin	1, 5, 30, 120	SBM	P	[52]
Phytase	0–1260	Wheat phytate globoids, wheat bran	InsP_6_ to InsP_2_	[53]
[60 min soaking phase]; β-glucanase; endo-xylanase; pepsin	0, 60	Corn, barley, wheat, potato protein concentrate, rapeseed expeller, soybean expeller	P	[54]

Abbreviations: InsP: InsP_6_: *myo*-inositol 1,2,3,4,5,6-hexakis (dihydrogen phosphate); InsP: inositol phosphate; InsP_3_: inositol phosphate_3_; InsP_2_: inositol phosphate_2_; SBM: soybean meal.

**Table 4:** Classification of feedstuffs by in vitro fermentation kinetic studies.

In Vitro Model			Feedstuff	Item	Reference
Basis of Assay	Enzymes	Sample Points (h)			
Undigested ingredients	Fecal inocula	0, 1, 2, 3, 4, 5, 6, 7, 8, 9, 10, 11, 12, 13, 14, 15, 16, 17, 18, 19, 20, 21, 22, 23, 24, 48	Inulin, lactulose, molasses-free sugar beet pulp, wheat starch	Gas production	[55]
Digested ingredients	Pepsin (120 min), pancreatin (240 min); fecal inocula	0, 2, 5, 8, 12, 18, 24, 36, 48	Wheat shorts, wheat millrun, wheat middlings, wheat bran	Gas production	[56]
Undigested ingredients	Fecal inoculum	0–72	Guar gum, konjac glucomannan, cellulose, retrograded tapioca starch, retrograded corn starch, oat β-glucan, inulin, oligofructose, HM citrus pectin, alginate, xanthan gum, soy pectin	Gas production	[57]
Digested ingredients	Pepsin (120 min), pancreatin (240 min); fecal inocula; *Trichoderma*-based carbohydrase (cellulase, xylanase) and/or protease *Bacillus* spp.	0, 2, 5, 8, 12, 18, 24, 36, 48, 72	Wheat DDGS, corn DDGS	Gas production	[58]
Digested ingredients	Pepsin (120 min), pancreatin (240 min); fecal inocula, xylanase, mannanase	0, 2, 5, 8, 12, 18, 24, 36, 48	Corn DDGS	Gas production	[59]
Digested ingredients	Pepsin, xylanase, glucanase, cellulase, mannanase, invertase, protease, amylase (120 min), pancreatin (240 min); fecal inocula;	0, 2, 5, 8, 12, 18, 24, 36, 48, 72	Corn wet distillers, corn DDGS Corn DDGS	Gas production	[60]
Undigested ingredients	Fecal inocula	0, 2, 5, 8, 12, 18, 24, 36, 48, 72	Rice starch (<45 g/kg DM amylose), rice starch (176 g/kg DM amylose), pea starch (256 g/kg DM amylose), corn starch (569 g/kg DM amylose)	Gas production	[61]
Undigested ingredients	Fecal inocula	0, 8, 16, 24, 32, 40, 48, 56	Wheat bran, soybean hulls, oat bran, corn bran, sugar beet pulp	Gas production	[62]

**Table 5:** Classification of feedstuffs or diets by in vitro or in vivo digestion or in vitro fermentation kinetics.

			Digestion Kinetics							Reference
In Vitro Model				Classification according to Sample Time (min)						
Basis of Assay	Enzymes	Sample Points (min)	Equation	Nutrient	Fast	Moderately Fast	Moderately Slow	Slow	Resistant	
Starch digestion kinetics										
Undigested ingredients	Amyloglucosidase; invertase; pancreatin; pullulanase; α-amylase	0, 20, 120	–	Glucose	0–20	–	–	20–120	>120; not further hydrolyzed	[67]
Undigested ingredients	Pepsin (30 min incubation); pancreatin; amyloglucosidase; invertase	0, 15, 30, 60, 120, 240, 360, 480	Chapman-Richards modified by [69]	Glucose	0–20	–	–	20–120	>120; not further hydrolyzed	[41] ^1^
Undigested ingredients	Pepsin (30 min incubation); pancreatin; amyloglucosidase; invertase	0, 15, 30, 60, 120, 240, 360, 480	Chapman-Richards modified by [69]	Starch hydrolyzed; glucose release	0–20	–	–	20–120	>120; not hydrolyzed	[68]
–	–	–	–	Starch hydrolysis in pigs	20 (digesta enters small intestine)	–	–	Difference starch fast hydrolyzed, and starch hydrolyzed at ileum	Not hydrolyzed at ileum	[68]
Protein digestion kinetics										
Undigested ingredients	Pepsin (90 min, pH 3.5); pancreatin; bile solution (pH 6.8)	0, 30, 60, 90, 120, 150, 180, 210	[70]; data fitted using linear equation	Nitrogen; low-molecular-weight peptides (<500 Da)	0–30	–	–	30–240	100–CP_fast_–CP_slow_	[38]
Undigested ingredients	Pepsin (pH 3.5); pancreatin; bile extract (pH 6.8)	0, 30, 60, 120, 240, 360	Gompertz equation	Nitrogen	0–30 min	–	–	30–240 min	100–CP_fast_–CP_slow_	[39]
Fiber fermentation kinetics										
Digested ingredients	Pepsin (120 min); pancreatin (240 min); fecal inocula	0, 2, 5, 8, 12, 18, 24, 36, 48, 72	[71]	Gas, CO_2_	L, 2.24; G_f_, 236	L, 2.38; G_f_, 226	L, 2.39; G_f_, 239	L, 2.67; G_f_, 219	–	[61]

Abbreviations: G_f_: total gas volume (mL per g sample incubated); L: lag time (h). ^1^ Data of in vitro assay corrected with predicted gastric emptying.

**Table 6:** Effect of fermentable substrates (starch, fiber, and protein) on the apparent ileal digestibility of minerals and InsP_6_ in growing pigs (initial body weight (**BW**) < 30 kg).

Initial BW (kg)	Diet Composition			Nutrient Composition of Diet (g/kg, as-Fed)							CAID (%)					Reference
	Main Ingredients	Fiber Source	Exogenous Enzymes	CP	P	InsP_6_-P	Ca/Zn	CF	NDF	ADF	CP	P	InsP_6_	Ca	Zn	
22	Sorghum, SBM	–	–	183	5.9	1.9	6.0	–	–	–	0.73	0.40	0.01	–	–	[141]
		–	Pancreatin								0.72	–	–	–	–	
		–	Phytase								0.72	0.52	0.36	–	–	
		–	Pancreatin, phytase								0.71	–	–	–	–	
28 ^1^	Corn, SBM	–	–	208	3.9	2.6	5.1	19	–	–	0.78 ^a^	0.29 ^aB^	0.31 ^b^	0.55 ^aB^	–	[113]
			Phytase	210	4.0	2.7	4.8	19	–	–	0.78 ^a^	0.62 ^aA^	0.92 ^a^	0.69 ^aA^	–	
		Rapeseed cake	–	178	4.9	3.5	6.1	31	–	–	0.71 ^b^	0.23 ^bA^	0.30 ^b^	0.47 ^bA^	–	
			Phytase	181	4.8	3.4	5.7	30	–	–	0.72 ^b^	0.57 ^bB^	0.92 ^a^	0.63 ^bB^	–	
28	Soy protein concentrate, fish meal	β-glucan, hull-less barley	–	246	7.4	3.2	8.5	–	260	23	–	0.07 ^b^	0.29	0.40	–	[135]
		Amylose, hull-less barley	–	210	7.7	2.7	7.8	–	145	23	–	0.16 ^b^	0.41	0.37	–	
		Hull-less barley	–	211	7.3	1.7	8.4	–	119	20	–	0.37 ^a^	0.26	0.53	–	
		Hulled barley	–	203	7.6	2.0	7.5	–	139	43	–	0.32 ^a^	0.13	0.41	–	
		Wheat	–	235	8.0	2.9	8.7	–	119	29	–	0.31 ^a^	0.19	0.38	–	

Abbreviations: ADF: acid detergent fiber; Ca: calcium; CP: crude protein; Cu: copper; InsP_6_: *myo*-inositol 1,2,3,4,5,6-hexakis (dihydrogen phosphate); InsP_6_-P: *myo*-inositol 1,2,3,4,5,6-hexakis (dihydrogen phosphate)-phosphorus; NDF: neutral detergent fiber; P: phosphorus; Zn: zinc. ^1^ Phytase (FTU/kg diet): <50, 1530, <50, 1370. ^a,b^ Within a row, means without a common superscript differ (*p* < 0.05). ^A,B^ Within a row, means without a common superscript differ (*p* < 0.05).

**Table 7:** Effect of fermentable substrates (starch, fiber, and protein) on apparent ileal digestibility of minerals and InsP_6_ in growing pigs (initial body weight (**BW**) > 30 kg).

Initial BW (kg)	Diet Composition			Nutrient Composition of Diet (g/kg, as-Fed)							CAID (%)					Reference
	Main Ingredients	Fiber Source	Exogenous Enzymes	CP	P	InsP_6_-P	Ca/Zn	CF	NDF	ADF	CP	P	InsP_6_	Ca	Zn	
40	Corn, barley, meat meal tankage	–	–	172	7.5	–	8.3	31	116	42	0.71 ^a^	0.28	–	0.33	–	[132]
		Pectin	–	165	7.1	–	8.0	30	110	40	0.70 ^ab^	0.25	–	0.26	–	
		Cellulose	–	164	7.1	–	7.9	58	156	83	0.70 ^ab^	0.28	–	0.28	–	
		Straw meal	–	165	7.2	–	7.9	50	148	64	0.67 ^b^	0.26	–	0.33	–	
85	Barley, corn starch, wheat, SBM	–	–	–	5.2	–	10.2/0.2	–	–	–	–	0.37	–	0.49	0.19	[133]
		Inulin	–	–	5.2	–	10.2/0.2	–	–	–	–	0.34	–	0.44	0.25	
36 ^1^	Wheat, SBM	–	–	–	6.4	2.7	7.4	–	–	–	–	0.54 ^a^	–	0.63 ^a^	–	[142]
		Wheat millrun (200 g/kg)	–	–	6.4	3.6	7.1	–	–	–	–	0.41 ^b^	–	0.54 ^b^	–	
			Xylanase	–							–	0.46	–	0.52	–	
			Phytase	–							–	0.43	–	0.55	–	
			Xylanase and phytase	–							–	0.48	–	0.48	–	
		Wheat millrun (400 g/kg)	–	–	6.2	4.5	6.8	–	–	–	–	0.35 ^c^	–	0.45 ^c^	–	
			Xylanase	–							–	0.38	–	0.41	–	
			Phytase	–							–	0.40	–	0.46	–	
			Xylanase and phytase	–							–	0.44	–	0.51	–	
36	Corn, SBM, corn starch	–	–	191	2.8	1.5	8.7	29	96	–	0.73 ^a^	0.26	0.60	0.59 ^a^	–	[130]
		Lignocellulose	–	149	2.1	1.2	6.8	200	311	–	0.65 ^ab^	0.25	0.60	0.62 ^a^	–	
		Corn starch	–	147	2.2	1.2	6.6	212	70	–	0.74 ^a^	0.15	0.18	0.62 ^a^	–	
		Pectin	–	147	2.3	1.1	6.9	219	73	–	0.48 ^b^	0.17	0.64	0.31 ^b^	–	
57	Corn starch	SBM	–	84.5	1.8	–	2.1	–	–	–	–	0.31 ^c^	–	–	–	[134]
			–	126	2.3	–	2.7	–	–	–	–	0.38 ^b^	–	–	–	
			–	167	3.0	–	3.4	–	–	–	–	0.41 ^a^	–	–	–	
		Canola meal	–	85.4	3.0	–	3.4	–	–	–	–	0.23	–	–	–	
			–	124	4.1	–	4.9	–	–	–	–	0.25	–	–	–	
			–	165	5.3	–	6.4	–	–	–	–	0.27	–	–	–	
		Corn DDGS	–	82.2	3.2	–	3.1	–	–	–	–	0.55	–	–	–	
			–	119	4.1	–	4.3	–	–	–	–	0.55	–	–	–	
			–	162	5.4	–	5.6	–	–	–	–	0.54	–	–	–	
	Corn	SBM	–	164	3.4	–	4.1	–	–	–	–	0.18 ^c^	–	–	–	
			–	158	4.5	–	5.3	–	–	–	–	0.31 ^b^	–	–	–	
			–	162	5.6	–	7.1	–	–	–	–	0.39 ^a^	–	–	–	
55 ^2^	Corn starch, sugar, bovine plasma protein	–	–	151	2.9	–	1.8	–	–	–	0.76	0.87	–	0.74	–	[124]
		Acacia gum (25 g/kg)	–	163	3.0	–	2.6	–	–	–	0.82	0.86	–	0.76	–	
		Acacia gum (50 g/kg)	–	155	2.9	–	2.7	–	–	–	0.79	0.82	–	0.72	–	
		Acacia gum (75 g/kg)	–	162	3.1	–	2.2	–	–	–	0.77	0.83	–	0.76	–	

Abbreviations: ADF: acid detergent fiber; Ca: calcium; CP: crude protein; Cu: copper; InsP_6_: *myo*-inositol 1,2,3,4,5,6-hexakis (dihydrogen phosphate); InsP_6_-P: *myo*-inositol 1,2,3,4,5,6-hexakis (dihydrogen phosphate)-phosphorus; NDF: neutral detergent fiber; P: phosphorus; Zn: zinc. ^1^ TDF, soluble, insoluble fiber (g/kg, as-fed): 114, 124, 159; 49.7, 30.9, 26.9; 64.9, 93.1, 132. ^2^ calculated TDF, soluble, insoluble fiber (g/kg, as-fed): 40.3, 59.9, 79.2, 98.9; 0.5, 19.8, 39.0, 58.1; 40.1, 40.3, 40.4, 40.5. ^a–c^ Within a row, means without a common superscript differ (*p* < 0.05).

**Table 8:** Effect of fermentable substrates (protein and fiber) on apparent total tract digestibility of minerals and InsP_6_ in weaned and growing pigs (initial body weight (**BW**) < 30 kg).

Initial BW (kg)	Diet Composition			Nutrient Composition (g/kg, as-Fed)							CATTD (%)					Reference
	Main Ingredients	Fiber Source	Exogenous Enzymes	CP	P	InsP_6_-P	Ca/Zn/Cu	CF	NDF	ADF	CP	P	InsP_6_	Ca	Zn	
29	Wheat	–	–	–	–	–	–	–	–	–	–	0.19 ^b^	–	–	–	[143]
		–	Xylanase	–	–	–	–	–	–	–	–	0.20 ^ab^	–	–	–	
		Wheat DDGS	–	–	–	–	–	–	–	–	–	0.49 ^a^	–	–	–	
			Xylanase	–	–	–	–	–	–	–	–	0.48 ^ab^	–	–	–	
8	Corn starch casein	–	–	204	3.3	0.5	6.1	12	–	–	–	0.42 ^b^	–	0.49 ^a^	–	[136]
		–	–	205	7.3	2.0	11	14	–	–	–	0.51 ^a^	–	0.46 ^b^	–	
		Oat β-glucan	–	220	3.3	1.3	6.0	14	–	–	–	0.27 ^b^	–	0.50 ^a^	–	
			–	216	8.3	2.8	9.6	13	–	–	–	0.65 ^a^	–	0.16 ^b^	–	
19 (Exp. 1) ^2^	Corn starch, sucrose, casein, fish meal	–	–	182	5.1	–	7.7	–	31	3.9	–	0.66 ^bB^	–	0.54 ^bB^	–	[137]
			Phytase	191	4.9	–	7.3	–	26	3.4	–	0.78 ^bA^	–	0.79 ^bA^	–	
	Corn, casein, fish meal	Corn germ	–	259	6.6	1.6	6.4	–	194	40	–	0.68 ^aB^	–	0.62 ^aB^	–	
			Phytase	270	7.0	1.6	7.0	–	211	60	–	0.75 ^aA^	–	0.71 ^aA^	–	
19 (Exp. 2)	Corn starch, sucrose, casein, fish meal	–		190	4.8	–	7.1	–	12	0.9	–	0.55 ^bB^	–	0.40 ^bB^	–	
		Cellulose	–	187	4.9	–	7.2	–	63	37	–	0.65 ^a^	–	0.57 ^a^	–	
	Corn, casein, fish meal, SB oil (10 g/kg)	–	–	237	6.4	1.3	7.4	–	71	24	–	0.76 ^A^	–	0.84 ^A^	–	
	Corn, casein, fish meal, SB oil (70 g/kg)	–	–	243	6.0	1.2	6.8	–	81	24	–	0.77	–	0.83	–	
25	Corn starch, sucrose, dextrose	Non-dehulled CM	–	232	3.5	–	2.9	–	117	–	–	0.32 ^b^	–	0.51 ^a^	–	[138]
		Dehulled CM	–	244	3.6	–	2.3	–	79	–	–	0.42 ^a^	–	0.55 ^a^	–	
		Coarse CM	–	232	3.2	–	2.2	–	117	–	–	0.25 ^c^	–	0.37 ^b^	–	
			phytase	153	4.7	–	6.0	–	131	73	–	0.46 ^a^	–	0.51	–	
11	Wheat, barley, SBM, lupine, corn starch, ZnSO_4_	Lignocellulose	–	182	5.0	2.0	NA/0.1/0.2	42	108	53	0.82	0.46 ^b^	0.89 ^a^	–	0.31 ^aB^	[131]
	Wheat, barley, SBM, lupine, corn starch, ZnGly		–	183	5.2	1.8	NA/0.1/0.2	40	107	56	0.83	0.45 ^b^	0.87 ^a^	–	0.28 ^bB^	
	Wheat, barley, SBM, lupine, ZnSO_4_	Potato fiber	–	184	5.1	1.7	NA/0.1/0.2	40	114	55	0.82	0.47 ^a^	0.86 ^b^	–	0.36 ^aA^	
	Wheat, barley, SBM, lupine, ZnGly		–	182	5.1	17	NA/0.1/0.2	40	109	58	0.82	0.50 ^a^	0.87 ^b^	–	0.32 ^bA^	
28 ^1^	Corn, SBM	–	–	208	3.9	2.6	5.1	19	–	–	0.89 ^a^	0.33 ^aB^	0.99	0.50 ^aB^	–	[113]
		–	Phytase	210	4.0	2.7	4.8	19	–	–	0.89 ^a^	0.64 ^aA^	0.99	0.68 ^aA^	–	
		Rapeseed cake	–	178	4.9	3.5	6.1	31	–	–	0.82 ^b^	0.24 ^bB^	0.99	0.42 ^bB^	–	
			Phytase	181	4.8	3.4	5.7	30	–	–	0.82 ^b^	0.52 ^bA^	0.99	0.58 ^A^	–	
28	Soy protein concentrate, fish meal	β-glucan, hull-less barley	–	246	7.4	32	8.5	–	260	23	–	0.47 ^b^	0.97 ^a^	0.48 ^ab^	–	[135]
		Amylose, hull-less barley	–	210	7.7	2.7	7.8	–	145	23	–	0.52 ^ab^	0.99 ^ab^	0.45 ^ab^	–	
		Hull-less barley	–	211	7.3	1.7	8.4	–	119	20	–	0.54 ^a^	0.96 ^ab^	0.50 ^ab^	–	
		Hulled barley	–	203	7.6	2.0	7.5	–	139	43	–	0.49 ^ab^	0.90 ^b^	0.36 ^b^	–	
		Wheat	–	235	8.0	2.9	8.7	–	119	29	–	0.52 ^ab^	0.97 ^ab^	0.55 ^a^	–	

Abbreviations: ADF: acid detergent fiber; Ca: calcium; CP: crude protein; Cu: copper; InsP_6_: *myo*-inositol 1,2,3,4,5,6-hexakis (dihydrogen phosphate); InsP_6_-P: *myo*-inositol 1,2,3,4,5,6-hexakis (dihydrogen phosphate)-phosphorus; NA: not analyzed; NDF: neutral detergent fiber; P: phosphorus; Zn: zinc. ^1^ Phytase (FTU/kg diet): <50, 1530, <50, 1370. ^2^ Phytase (FTU/kg diet): not detected, 667, not detected, 712. ^a–c^ Within a row, means without a common superscript differ (*p* < 0.05). ^A,B^ Within a row, means without a common superscript differ (*p* < 0.05).

**Table 9:** Effect of fermentable substrates (protein and fiber) on apparent total tract digestibility of minerals and InsP_6_ in growing pigs (initial body weight (**BW**) > 30 kg).

Initial BW (kg)	Diet Composition			Nutrient Composition (g/kg, as-Fed)							CATTD (%)					Reference
	Main Ingredients	Fiber Source	Exogenous Enzymes	CP	P	InsP_6_-P	Ca/Zn/Cu	CF	NDF	ADF	CP	P	InsP_6_	Ca	Zn	
40	Corn, barley, meat meal tankage	–	–	172	7.5	–	8.3	31	116	42	0.85	0.42	–	0.43 ^a^	–	[132]
		Pectin	–	165	7.1	–	8.0	30	110	40	0.83	0.46	–	0.39 ^ab^	–	
		Cellulose	–	164	7.1	–	7.9	58	156	83	0.82	0.42	–	0.35 ^b^	–	
		Straw meal	–	165	7.2	–	7.9	50	148	64	0.83	0.44	–	0.39 ^ab^	–	
85	Barley, wheat, corn starch, SBM	–	–	–	5.2	–	10.2/0.2	–	–	–	–	0.32	–	0.39	0.05	[133]
		Inulin	–	–	5.2	–	10.2/0.2	–	–	–	–	0.29	–	0.37	0.11	
65	Wheat	–	–	–	–	–	–	–	–	–	–	0.15 ^b^	–	–	–	[144]
		Corn DDGS	–	–	–	–	–	–	–	–	–	0.56 ^a^	–	–	–	
		Wheat/corn DDGS	–	–	–	–	–	–	–	–	–	0.55 ^a^	–	–	–	
		Wheat DDGS	–	–	–	–	–	–	–	–	–	0.53 ^a^	–	–	–	
36 ^1^	Wheat, SBM	–	–	–	6.4	2.7	7.4	–	–	–	–	0.60 ^a^	–	0.62 ^a^	–	[142]
		Wheat millrun (200 g/kg)	–	–	6.4	3.6	7.1	–	–	–	–	0.45 ^bB^	–	0.54 ^b^	–	
			Xylanase	–							–	0.48	–	0.55	–	
			Phytase	–							–	0.52 ^A^	–	0.56	–	
			Xylanase and phytase	–							–	0.60 ^A^	–	0.57	–	
		Wheat millrun (400 g/kg)	–	–	6.2	4.5	6.8	–	–	–	–	0.43 ^c^	–	0.45 ^c^	–	
			Xylanase	–							–	0.44	–	0.45	–	
			Phytase	–							–	0.46 ^A^	–	0.50	–	
			Xylanase and phytase	–							–	0.54 ^A^	–	0.48	–	
36	Corn, SBM, corn starch	–	–	191	2.8	1.5	8.7	29	96	–	0.87 ^a^	0.28 ^a^	–	0.60 ^a^	–	[130]
		Lignocellulose	–	149	2.1	1.2	6.8	200	311	–	0.75 ^b^	0.24 ^ab^	–	0.61 ^a^	–	
		Corn starch	–	147	2.2	1.2	6.6	212	70	–	0.89 ^a^	0.23 ^ab^	–	0.56 ^a^	–	
		Pectin	–	147	2.3	1.1	6.9	219	73	–	0.76 ^b^	0.15 ^b^	–	0.30 ^b^	–	
			–	216	8.3	2.8	9.6	13	–	–	–	0.65 ^a^	–	0.16 ^b^	–	
35–40 ^2^	Wheat, barley, SBM	–	–	194	3.6	–	NA/0.05	–	–	–	–	0.40 ^b^	–	–	0.28 ^a^	[145]
	+formic acid	–	–	192	3.6	–	NA/0.04	–	–	–	–	0.33 ^b^	–	–	0.12 ^b^	
	Wheat, barley, SBM	–	Phytase (500 FTU)	197	3.7	–	NA/0.04	–	–	–	–	0.52 ^a^	–	–	0.26 ^a^	
	+formic acid	–	Phytase (500 FTU)	196	3.7	–	NA/0.04	–	–	–	–	0.60 ^a^	–	–	0.27 ^a^	
35–40	Wheat, barley, SBM	–	–	194	3.6	–	NA/0.05	–	–	–	–	0.41 ^b^			0.27 ^ab^	
	+formic acid	–	–	192	3.6	–	NA/0.04	–	–	–	–	0.39 ^b^			0.20 ^b^	
	Wheat, barley, SBM	–	Phytase (1000 FTU)	195	3.6	–	NA/0.04	–	–	–	–	0.62 ^a^	–	–	0.32 ^a^	
	+formic acid	–	Phytase (1000 FTU)	191	3.6	–	NA/0.04	–	–	–	–	0.67 ^a^	–	–	0.35 ^a^	
33	Potato starch, beet pulp	Wheat bran	–	137	3.3	–	5.0	42	–	–	0.80	0.43 ^b^	–	0.59 ^b^	–	[146]
		Wheat bran, fermented	–	135	3.3	–	5.1	42	–	–	0.82	0.58 ^a^	–	0.65 ^a^	–	
		Wheat bran, extruded	–	139	3.5	–	5.1	37	–	–	0.82	0.38 ^b^	–	0.51 ^c^	–	
57	Corn starch	SBM	–	84.5	1.8	–	2.1	–	–	–	–	0.35 ^c^	–	–	–	[134]
			–	126	2.3	–	2.7	–	–	–	–	0.42 ^b^	–	–	–	
			–	167	3.0	–	3.4	–	–	–	–	0.45 ^a^	–	–	–	
		Canola meal	–	85.4	3.0	–	3.4	–	–	–	–	0.27	–	–	–	
			–	124	4.1	–	4.9	–	–	–	–	0.29	–	–	–	
			–	165	5.3	–	6.4	–	–	–	–	0.30	–	–	–	
		Corn DDGS	–	82.2	3.2	–	3.1	–	–	–	–	0.65	–	–	–	
			–	119	4.1	–	4.3	–	–	–	–	0.67	–	–	–	
			–	162	5.4	–	5.6	–	–	–	–	0.66	–	–	–	
	Corn	SBM	–	164	3.4	–	4.1	–	–	–	–	0.27 ^c^	–	–	–	
			–	158	4.5	–	5.3	–	–	–	–	0.43 ^b^	–	–	–	
			–	162	5.6	–	7.1	–	–	–	–	0.50 ^a^	–	–	–	
79	Corn starch, sucrose, dextrose	Non-dehulled CM	–	191	3.6	–	2.9	–	99	–	–	0.31 ^ab^	–	0.44 ^a^	–	
		Dehulled CM	–	203	3.7	–	2.5	–	54	–	–	0.39 ^a^	–	0.28 ^b^	–	
		Coarse CM	–	205	3.5	–	2.5	–	88	–	–	0.23 ^b^	–	0.44 ^a^	–	
36 ^3^	Corn, SBM	Rapeseed meal	–	154	4.7	–	4.4	–	105	51	–	0.30	–	0.50	–	[147]
			Phytase	146	4.4	–	6.2	–	105	51	–	0.43 ^a^	–	0.51	–	
		Dehulled rapeseed meal	–	146	4.4	–	4.0	–	78	29	–	0.26	–	0.50	–	
			Phytase	145	4.3	–	5.8	–	78	29	–	0.45 ^a^	–	0.53	–	
		Dehulled rapeseed meal, rapeseed hulls (45 g/kg)	–	147	4.5	–	4.4	–	100	53	–	0.24	–	0.48	–	
			Phytase	147	4.6	–	5.8	–	100	53	–	0.46 ^a^	–	0.53	–	
		Dehulled rapeseed meal, rapeseed hulls (90 g/kg)	–	152	4.6	–	4.2	–	131	73	–	0.25	–	0.51	–	
			Phytase	153	4.7	–	6.0	–	131	73	–	0.46 ^a^	–	0.51	–	
55	Corn, SBM	–	–	152	3.7	1.9	3.9	–	125	59	0.74	0.02 ^Bb^	0.09	0.45 ^a^	–	[115]
		–	–	151	6.8	1.8	7.5	–	124	52	0.75	0.21 ^Ba^	0.14	0.35 ^b^	–	
	Corn	Field pea	–	138	3.8	1.8	4.1	–	92	53	0.69	0.05 ^Ab^	0.13	0.43 ^a^	–	
			–	133	6.6	1.8	7.5	–	96	58	0.74	0.24 ^Aa^	0.10	0.36 ^b^	–	
55 ^4^	Corn starch, sugar, bovine plasma protein	–	–	151	2.9	–	1.8	–	–	–	0.95	0.88	–	0.74	–	[124]
		Acacia gum (25 g/kg)	–	163	3.0	–	2.6	–	–	–	0.95 ^a^	0.86	–	0.71	–	
		Acacia gum (50 g/kg)	–	155	2.9	–	2.7	–	–	–	0.95 ^b^	0.83	–	0.79	–	
		Acacia gum (75 g/kg)	–	162	3.1	–	2.2	–	–	–	0.94 ^c^	0.81	–	0.78	–	

Abbreviations: ADF: acid detergent fiber; Ca: calcium; CP: crude protein; Cu: copper; InsP_6_: *myo*-inositol 1,2,3,4,5,6-hexakis (dihydrogen phosphate); InsP_6_-P: *myo*-inositol 1,2,3,4,5,6-hexakis (dihydrogen phosphate)-phosphorus; NA: not analyzed; NDF: neutral detergent fiber; P: phosphorus; Zn: zinc. ^1^ TDF, soluble, insoluble fiber (g/kg, as-fed): 114, 124, 159; 49.7, 30.9, 26.9; 64.9, 93.1, 132. ^2^ Phytase (FTU/kg diet): < 100, < 100, 600, 620, < 100, < 100, 1240, 1270. ^3^ Phytase (FTU/kg diet): <100, 650, <100, 450, <450, <100, 870, <100, 630. ^4^ calculated TDF, soluble, insoluble fiber (g/kg, as-fed): 40.3, 59.9, 79.2, 98.9; 0.5, 19.8, 39.0, 58.1; 40.1, 40.3, 40.4, 40.5. ^a–c^ Within a row, means without a common superscript differ (*p* < 0.05). ^A,B^ Within a row, means without a common superscript differ (*p* < 0.05).

## Data Availability

Not applicable.
